# Effectiveness of Robot-Assisted Gait Training in Stroke Rehabilitation: A Systematic Review and Meta-Analysis

**DOI:** 10.3390/jcm14134809

**Published:** 2025-07-07

**Authors:** Jun Hyeok Lee, Gaeun Kim

**Affiliations:** 1 Graduate School of Education, Keimyung University, 1095 Dalgubeol-daero, Dalseo-gu, Daegu 42601, Republic of Korea; jekfl@naver.com; 2 Namsan Hospital, 9 Namsan-ro 13-gil, Jung-gu, Daegu 41978, Republic of Korea; 3 College of Nursing, Keimyung University, 1095 Dalgubeol-daero, Dalseo-gu, Daegu 42601, Republic of Korea

**Keywords:** stroke, robotic-assisted gait training, rehabilitation, gait function, balance, activities of daily living, meta-analysis

## Abstract

**Background/Objectives:** Robotic-assisted gait training (RAGT) is a promising adjunct to conventional rehabilitation for stroke survivors. However, its additive benefit over standard therapy remains to be fully clarified. This systematic review and meta-analysis evaluated the effectiveness of combining RAGT with conventional rehabilitation in improving gait-related outcomes among individuals with stroke. **Methods:** We searched PubMed, Embase, CINAHL, and Cochrane CENTRAL through September 2024 for randomized controlled trials (RCTs) comparing combined RAGT and conventional rehabilitation versus conventional rehabilitation alone in adults post-stroke. Data were synthesized using a random-effects model, and subgroup analyses examined effects by intervention duration, stroke chronicity, and robotic system type. **Results:** Twenty-three RCTs (n = 907) were included. The combined intervention significantly improved gait function (SMD = 0.51, *p* = 0.001), gait speed (SMD = 0.47, *p* = 0.010), balance (MD = 4.58, *p* < 0.001), and ADL performance (SMD = 0.35, *p* = 0.001). Subgroup analyses revealed that end-effector robotic systems yielded superior outcomes compared to exoskeletons, particularly in subacute stroke patients. The most pronounced benefits were seen in gait velocity and dynamic balance, especially with ≤15 training sessions. **Conclusions:** Integrating RAGT with conventional rehabilitation enhances motor recovery and functional performance in stroke survivors. End-effector devices appear most effective in subacute phases, supporting individualized RAGT application based on patient and device characteristics.

## 1. Introduction

Stroke, resulting from cerebral ischemia or hemorrhage, is a leading cause of adult disability worldwide, producing a wide range of neurological impairments depending on the location and extent of brain injury [1]. In South Korea, over 100,000 new stroke cases are reported annually, with incidence steadily increasing among individuals aged 65 years and older due to demographic aging [2]. Among the various sequelae, gait dysfunction is particularly debilitating, as it impairs independence in daily living, restricts social participation, and substantially lowers quality of life. Large-scale cohort studies have identified post-stroke gait deficits as strong predictors of long-term disability, reduced community reintegration, and elevated caregiver burden [3].

Globally, stroke-related gait impairments are among the leading causes of mobility restrictions and fall-related injuries in older adults, particularly in aging societies such as Japan, Italy, and the United States [4,5].

Functional ambulation is a cornerstone of stroke recovery and a key target of rehabilitation. For instance, a multicenter cohort study involving 1475 stroke survivors reported that slower gait speed within two weeks post-onset predicted significantly poorer health-related quality of life at 3 and 12 months, especially in mobility, self-care, and usual activities [6]. Elevated gait variability has also been associated with impaired balance and increased fall risk in chronic stroke populations [7]. Conventional rehabilitation programs—comprising treadmill walking, strength training, and dynamic balance exercises—are central to improving gait. Nurses play a vital role in these programs by promoting adherence, assisting in ambulation and transfers, monitoring physical and psychological responses, and providing patient education to reinforce engagement. Despite these efforts, real-world implementation is often limited by therapist shortages, variable training intensity, and inconsistent session delivery.

Robotic-assisted gait training (RAGT) has emerged as a scalable and technology-enhanced intervention designed to address these limitations. RAGT systems enable high-intensity, repetitive, and programmable gait training with standardized movement patterns tailored to individual capabilities [8]. These systems facilitate symmetrical gait cycles and can reduce therapist burden while maintaining consistent training quality [9]. RAGT has gained traction in stroke rehabilitation worldwide and has been increasingly implemented in South Korea since 2022. However, practical adoption is still constrained by high costs, equipment availability, and infrastructural demands. Furthermore, evidence supporting RAGT efficacy remains heterogeneous, with previous meta-analyses incorporating various robotic and non-robotic technologies—such as virtual reality or functional electrical stimulation—thus complicating conclusions specific to RAGT [9].

In particular, recent neurophysiological theories suggest that robotic systems may facilitate recovery through enhanced sensorimotor feedback and structured repetition, activating latent motor learning capacity even in chronic stroke survivors [10,11]. Stroke recovery can be categorized into acute (<7 days), subacute (7 days–6 months), and chronic (>6 months) phases, and response to interventions may differ accordingly [12]. Clinical guidelines highlight the importance of intensive, task-specific, and repetitive training to stimulate neuroplasticity [13]. However, previous studies have not systematically evaluated how intervention characteristics—such as robot type (end-effector vs. exoskeleton), stroke chronicity, and total training sessions—may moderate treatment effects [14,15,16,17,18]. In addition, it remains unclear which functional components—such as gait coordination, postural stability, or sensorimotor integration—are specifically enhanced by robotic systems compared to conventional care provided solely by nurses.

Standardized outcome measures—such as gait function, gait speed, balance (e.g., BBS, TUG), and activities of daily living (e.g., FIM)—are commonly used to assess motor and functional recovery. These domains are critical for functional independence and guide rehabilitation goal-setting.

Therefore, this systematic review and meta-analysis aimed to evaluate the effectiveness of RAGT combined with conventional rehabilitation compared to conventional rehabilitation alone in stroke survivors. The primary outcomes included gait function, gait speed, balance, and ADLs. Subgroup analyses were conducted based on robot type, total number of intervention sessions, and stroke phase to identify potential moderators of treatment effectiveness. The findings are expected to inform evidence-based practice and clarify how RAGT can complement traditional nursing roles in post-stroke care.

## 2. Methods

### 2.1. Study Design

This systematic review and meta-analysis was designed to evaluate the effectiveness of conventional rehabilitation combined with RAGT in adult stroke patients. The protocol was developed in accordance with the PRISMA 2020 guidelines and the Cochrane Handbook for Systematic Reviews of Interventions [19].

### 2.2. Eligibility Criteria

Studies were selected using the Population, Intervention, Comparison, Outcome, and Study Design (PICOS) framework. Eligible studies met the following criteria: (1) adult participants (aged ≥ 18 years) with ischemic or hemorrhagic stroke; (2) interventions combining conventional lower-limb rehabilitation with RAGT (exoskeleton or end-effector systems); (3) control groups receiving only conventional rehabilitation; (4) reported outcomes on gait function, gait speed, gait balance, or ADLs; and (5) randomized controlled trial (RCT) design with extractable effect size data. Exclusion criteria included studies involving pediatric participants, robotic-only interventions without concurrent conventional therapy, unequal session durations between groups, or non-RCT formats such as reviews, conference abstracts, or case reports.

### 2.3. Search Strategy

A comprehensive search was conducted in PubMed, Embase, CINAHL, and the Cochrane CENTRAL from inception to 30 September 2024. No language filters were applied. Search strategies utilized Medical Subject Headings (MeSH), EMTREE terms, and free-text keywords. A comprehensive search strategy was developed using both controlled vocabulary (e.g., MeSH, EMTREE) and free-text terms across all databases. Boolean operators (AND, OR) and truncations were applied to optimize sensitivity. A representative PubMed query included: “(Stroke[MeSH] OR cerebrovascular accident OR CVA OR CVD) AND (Robotics[MeSH] OR robotic-assisted gait training OR RAGT OR exoskeleton OR end-effector) AND (Rehabilitation[MeSH] OR physical therapy OR gait training).”

### 2.4. Study Selection and Data Extraction

Citations were imported into EndNote 20, and duplicates were removed. Two independent reviewers screened titles and abstracts and subsequently reviewed full texts against inclusion criteria. Discrepancies were resolved through discussion. Data extraction employed a Cochrane-based standardized form. Extracted data included author/year, study design and setting, participant demographics (sample size, age, stroke duration), intervention details (robot type, duration, frequency), comparator characteristics, outcome measures (e.g., gait speed, ADLs), and pre/post-means and standard deviations.

When studies reported medians and interquartile ranges (IQRs), means and standard deviations were calculated using the formulas proposed by Wan et al. [20]. These converted values were then used to compute standardized mean differences (SMDs). Supplementary Table S1 provides the list of all outcome measures, their original scales, score ranges, directionality, and clinical interpretations.

### 2.5. Risk of Bias Assessment

The risk of bias for each included randomized controlled trial was independently assessed by two reviewers using the Cochrane Risk of Bias 2.0 (RoB 2.0) tool [21], which evaluates domains such as randomization process, deviations from intended interventions, missing outcome data, measurement of the outcome, and selection of the reported result. Discrepancies were resolved through discussion.

### 2.6. Data Analysis

All statistical analyses were performed using Review Manager (RevMan) version 5.4.1 [22]. Random-effects models were used to account for inter-study variability. Standardized mean differences (SMDs) with 95% confidence intervals (CIs) were computed for continuous outcomes. If studies reported medians and interquartile ranges, these were converted to means and standard deviations using validated methods. Gait speed was standardized to meters/second. When studies reported medians and interquartile ranges, means and standard deviations were calculated using the formulas proposed by Wan et al. [20].

Heterogeneity was quantified using the I^2^ statistic, with thresholds of 0–25%, 26–50%, 51–75%, and >75% representing low to very high heterogeneity [23]. Subgroup analyses were predefined based on clinical relevance and potential sources of heterogeneity. Categories included robot type (end-effector vs. exoskeleton), training frequency (≤15 vs. >15 sessions), and stroke chronicity (acute, subacute, chronic). Random-effects meta-regression models were applied to estimate subgroup-specific effect sizes. Interaction *p*-values were used to assess differences between subgroups.

Sensitivity analyses were conducted by excluding studies with high risk of bias and those with imputed values to assess the robustness of pooled estimates. Potential publication bias was examined via funnel plot symmetry for outcomes with ≥10 studies. Egger’s regression test was also conducted, with non-significant results and symmetric plots indicating a low risk of bias.

## 3. Result

### 3.1. Study Selection

**Figure 1:**
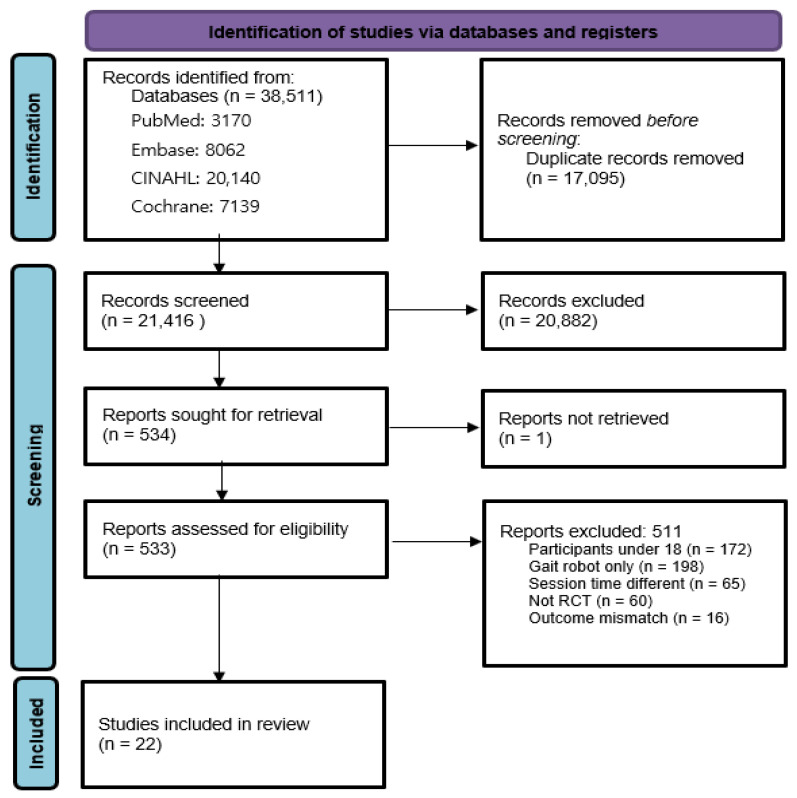
PRISMA flowchart.

A total of 38,511 records were initially identified through systematic database searches. After removing 17,095 duplicates, 21,416 unique titles and abstracts were screened. Among these, 511 studies underwent full-text assessment, and 489 were excluded for not meeting inclusion criteria. Ultimately, 22 RCTs were selected [1,12,16,24,25,26,27,28,29,30,31,32,33,34,35,36,37,38,39,40,41,42]. One study [28] included two distinct subgroups based on baseline motor ability (high vs. low), which were analyzed independently. This classification was specific to that study and not part of the predefined subgroup analyses of robot type, intervention session count, and stroke chronicity (Figure 1).

### 3.2. Characteristics of Included Studies

The 23 included datasets comprised 907 patients, with a mean age ranging from 44 to 76 years. Most studies targeted individuals in the subacute phase of stroke (n = 17), with ischemic stroke being slightly more prevalent than hemorrhagic types. Eleven studies used end-effector robots, while eleven used exoskeletons. Intervention duration ranged from 2 to 12 weeks, most frequently conducted 5 times per week. These characteristics are summarized in Table 1. The included studies comprised 907 stroke patients (524 ischemic and 383 hemorrhagic). Study publication years ranged from 2005 to 2024. Robotic systems were classified as either exoskeleton (n = 11) or end-effector (n = 11). Intervention frequency was most commonly five times per week (n = 15), and a four-week duration was typical (n = 9). The most common total number of sessions was 20 (n = 11). Baseline characteristics, intervention parameters, and outcomes varied slightly across studies.

Rehabilitation tasks included gait training, weight shifting, balance control, and functional ambulation exercises. Robot engagement ranged from 20 to 60 min per session, with most protocols implemented in inpatient hospital settings. End-effector systems were more commonly used in subacute cases and were associated with more frequent training sessions.

### 3.3. Risk of Bias Assessment

**Figure 2:**
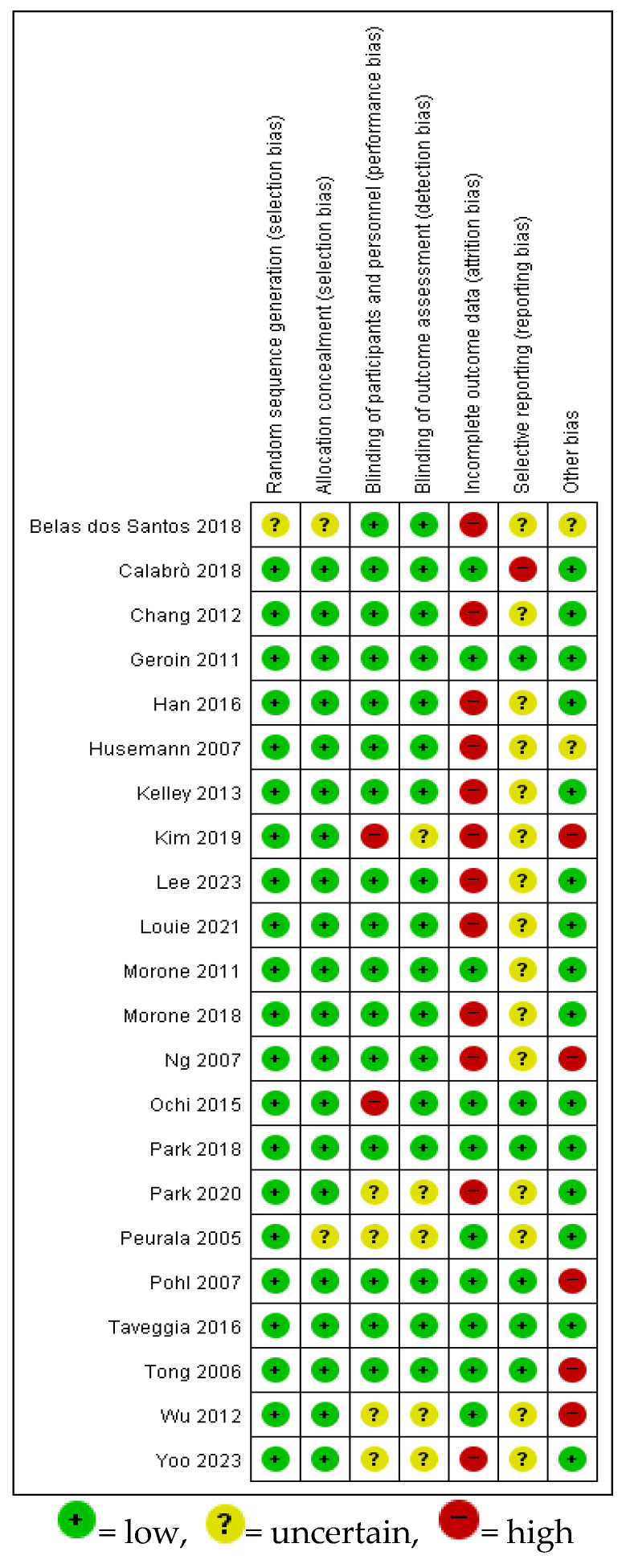
Results of risk of bias assessment of the selected literature [1,12,16,24,25,26,27,28,29,30,31,32,33,34,35,36,37,38,39,40,41,42].

Most studies reported adequate randomization and allocation concealment. Blinding of outcome assessors was well addressed in the majority. Overall, selection, performance, and detection bias were considered low to moderate, indicating acceptable methodological quality (Figure 2). Most studies reported adequate random sequence generation and allocation concealment. Although participant and therapist blinding was generally not feasible due to the nature of the intervention, the blinding of outcome assessors was described in a majority of trials. Furthermore, outcome reporting was generally complete, with few instances of missing data across the included studies.

### 3.4. Effects of Combined Robotic and Conventional Gait Rehabilitation

#### 3.4.1. Gait Function

**Figure 3:**
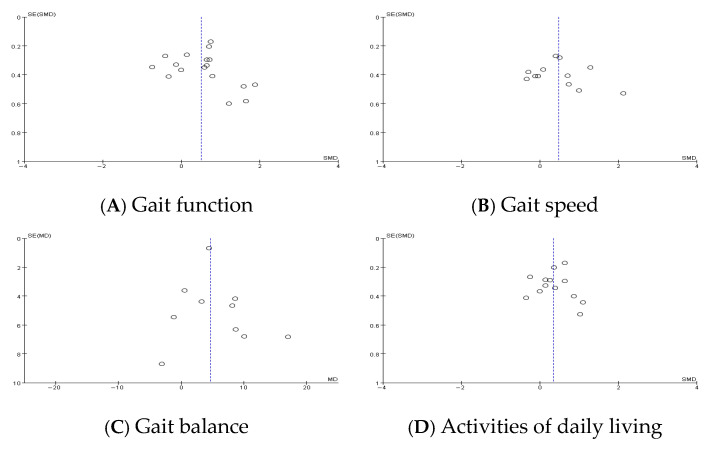
Publication bias.

Seventeen studies assessed gait function using validated scales (e.g., Functional Ambulation Category, FAC). Meta-analysis revealed a significant benefit in the intervention group (SMD = 0.51, 95% CI: 0.21–0.81, *p* = 0.001), though heterogeneity was high (I^2^ = 73%). Subgroup analysis showed that end-effector robots (n = 10) produced more consistent effects (SMD = 0.53, 95% CI: 0.28–0.79, I^2^ = 51%, *p* < 0.001) compared to exoskeleton robots (n = 7; SMD = 0.55, 95% CI: –0.19 to 1.30, I^2^ = 84%, *p* = 0.150) (Table 2, Figure 3A).

#### 3.4.2. Gait Speed

Twelve studies provided gait speed data. The pooled effect was statistically significant (SMD = 0.47, 95% CI: 0.12–0.83, *p* = 0.010, I^2^ = 62%). Subgroup analysis by stroke phase revealed a modest effect in subacute patients (n = 7; SMD = 0.31, 95% CI: 0.00–0.61, *p* = 0.050, I^2^ = 23%) and a larger but non-significant effect in chronic patients (n = 5; SMD = 0.76, 95% CI: −0.04 to 1.55, *p* = 0.060, I^2^ = 79%). End-effector robots (n = 6) showed significant improvement (SMD = 0.30, 95% CI: 0.03–0.58, *p* = 0.030, I^2^ = 0%), while exoskeletons showed a trend (SMD = 0.72, 95% CI: −0.01 to 1.45, *p* = 0.050, I^2^ = 78%) (Figure 3B).

#### 3.4.3. Gait Balance

Ten studies assessed balance using the Berg Balance Scale (BBS). The pooled mean difference was 4.58 points (95% CI: 3.03–6.13, *p* < 0.001), with very low heterogeneity (I^2^ = 2%). This reflects a consistent improvement across studies in postural stability and functional balance (Figure 3C).

#### 3.4.4. Activities of Daily Living (ADLs)

Thirteen studies evaluated ADL outcomes using indices such as the Barthel Index. The pooled standardized mean difference was 0.35 (95% CI: 0.14–0.56, *p* = 0.001, I^2^ = 39%). This moderate effect indicates improved functional independence in daily tasks among patients receiving RAGT (Figure 3D).

In the intervention groups, robotic systems primarily supported high-repetition locomotor training by automating symmetrical gait cycles, providing adjustable cadence and resistance levels, and facilitating balance through trunk and limb support. These features allowed for consistent and intensive gait practice with minimal therapist fatigue. In contrast, in the control groups (conventional rehabilitation only), physical therapists and nurses manually guided patients through similar tasks with more variable intensity and timing. In both settings, nurses played crucial roles in safety monitoring, patient encouragement, and physical assistance during transfers or postural adjustments. The division of labor into ‘robot plus nurse’ teams enabled more structured task allocation, potentially enhancing treatment fidelity and patient adherence.

#### 3.4.5. Publication Bias

Funnel plot asymmetry was observed for gait function outcomes, suggesting potential publication bias. This was supported by Egger’s regression test, which yielded a statistically significant intercept (2.41, *p* = 0.023). In contrast, funnel plots for gait speed, balance, and activities of daily living (ADLs) appeared symmetrical, indicating a lower risk of publication bias for these outcomes. Sensitivity analysis excluding studies with a high risk of bias did not significantly alter the pooled estimates, confirming the robustness of the findings.

Funnel plots were generated to visually assess potential publication bias in each outcome domain. The plot for gait function demonstrated notable asymmetry, suggesting a small-study effect or selective outcome reporting. In contrast, the plots for gait speed, balance, and ADLs showed relatively symmetrical distributions. These graphical patterns are consistent with the statistical findings and support the validity of the synthesized results, especially for the latter three outcomes.

## 4. Discussion

This systematic review and meta-analysis evaluated the effectiveness of robotic-assisted gait training (RAGT) as an adjunct to conventional rehabilitation in adult stroke survivors. The pooled results demonstrated significant improvements in gait function (SMD = 0.51), gait speed (SMD = 0.47), balance (MD = 4.58), and activities of daily living (ADLs; SMD = 0.35). These findings support the clinical utility of RAGT in enhancing post-stroke functional recovery.

Compared to conventional rehabilitation alone, RAGT offers distinct advantages by delivering high-frequency, symmetrical, and task-specific gait cycles. These repetitive and standardized movement patterns facilitate motor relearning and promote neuroplasticity, which is essential for cortical reorganization after stroke. Stroke survivors often experience persistent deficits in coordination and balance that impede mobility and quality of life [5], and the precision of robotic systems helps address these impairments more consistently than manual therapy alone.

Subgroup analyses revealed that end-effector robots were associated with more consistent improvements in gait speed (SMD = 0.30, I^2^ = 0%) than exoskeleton robots, which showed greater variability (SMD = 0.72, I^2^ = 78%). This aligns with a recent Korean RCT reporting significant gait gains in subacute stroke patients using end-effector systems [1]. The superior performance of end-effector devices may be attributed to their ability to provide naturalistic gait trajectories and plantar sensory stimulation, enhancing proprioceptive input and sensorimotor integration [43]. Studies by Calabrò et al. [25] further support this by demonstrating increased cortical connectivity and neuroplastic changes following RAGT incorporating sensory feedback.

Balance improvement, as measured by the Berg Balance Scale (BBS), was clinically meaningful, exceeding the minimal detectable change (MDC) of 4–5 points [44]. Enhanced balance reduces fall risk—a key contributor to post-stroke morbidity and dependency—and may improve psychosocial outcomes by reducing fear of falling [45]. Similarly, improvements in ADLs, though modest, suggest broader functional benefits that may reduce caregiver burden and improve discharge readiness and community reintegration.

However, not all functional domains responded equally to RAGT. Several studies reported limited improvement in ADLs despite significant gains in motor performance. This discrepancy may reflect the multifactorial nature of functional independence, which is shaped not only by physical ability but also by cognitive status, motivation, environmental context, and psychosocial support. These findings underscore the need for multimodal rehabilitation strategies that combine RAGT with behavioral, cognitive, and environmental interventions to optimize real-world functional recovery [14].

Interestingly, some included studies reported greater improvements among patients in the chronic phase compared to those in the subacute phase, which appears counterintuitive. This finding may be interpreted through the lens of the motor learning reserve hypothesis, suggesting that individuals with longstanding motor impairments may still retain the capacity for functional gains when provided with highly structured, repetitive interventions. Robotic systems may serve as consistent proprioceptive stimulators that elicit neuromuscular adaptation even in later recovery stages, thereby supporting neuroplasticity in chronic stroke survivors.

Another important finding is the moderating role of stroke chronicity in treatment response. Patients in the subacute phase demonstrated greater gains than those in the chronic phase, consistent with existing evidence that the brain’s neuroplastic potential is highest in the early stages post-stroke. This highlights the importance of early intervention and supports the development of phase-specific rehabilitation algorithms that align RAGT intensity and duration with patients’ recovery windows. Although intervention durations across the included studies ranged from 2 to 12 weeks, most were clustered between 4 and 8 weeks. However, long-term follow-up was rarely conducted, limiting conclusions about the sustainability of observed improvements. Future trials should incorporate extended follow-up periods and investigate how RAGT protocols might be adapted based on the stroke phase to optimize long-term functional outcomes and guide personalized rehabilitation planning.

From a healthcare system perspective, RAGT represents a scalable solution to address workforce shortages and the increasing demand for rehabilitation services in aging populations. By improving standardization and training intensity, RAGT may enhance recovery efficiency, reduce long-term disability, and support independent living. Nonetheless, barriers such as high costs, limited device availability, and the need for trained personnel must be addressed. Economic evaluations are therefore essential to determine cost-effectiveness and support equitable policy implementation.

In the robot-assisted intervention groups, the robotic systems primarily assumed tasks involving lower-limb movement generation, trunk stabilization, and gait cycle regulation. This reduced the need for physical manual guidance by nurses or therapists, allowing them to redirect focus toward safety supervision, psychological support, and individualized patient education. In contrast, conventional rehabilitation required more direct physical involvement from nurses. Such division of labor suggests that the future implementation of RAGT may not only enhance rehabilitation fidelity but also alleviate caregiver burden, paving the way for optimized interdisciplinary teamwork.

Finally, this review is not without limitations. Potential publication bias was noted based on funnel plot asymmetry and Egger’s test, though trim-and-fill analysis was not performed due to the small number of studies per outcome. Furthermore, moderate-to-high heterogeneity in several outcomes may reflect differences in robotic device types, intervention durations, and patient characteristics. Most included trials could not blind participants or therapists, which increases the risk of performance bias. Nevertheless, the frequent use of blinded outcome assessors and adequate randomization procedures improves the internal validity of the findings.

In summary, this review provides robust evidence that RAGT is an effective, technology-enhanced intervention for improving motor and functional outcomes in stroke rehabilitation. Its optimal use depends on stroke phase, robot type, and patient-specific factors, highlighting the importance of individualized and evidence-informed clinical pathways.

## 5. Conclusions

This systematic review and meta-analysis demonstrates that robot-assisted gait training (RAGT), when integrated with conventional rehabilitation, significantly improves functional outcomes in stroke survivors. Specifically, the combined intervention was associated with meaningful improvements in gait function (SMD = 0.51, *p* = 0.001), gait speed (SMD = 0.47, *p* = 0.010), balance (MD = 4.58, *p* < 0.001), and activities of daily living (SMD = 0.35, *p* = 0.001) compared to conventional therapy alone. Among the different types of robotic devices, end-effector robots produced more consistent benefits, which may be attributed to their ability to facilitate natural stepping patterns and deliver enhanced sensory feedback.

These findings support the clinical value of RAGT as an effective adjunct to conventional post-stroke rehabilitation. However, the variation in patient characteristics, stroke chronicity, and intervention protocols across the included studies highlights the need for individualized approaches. To enhance clinical effectiveness, rehabilitation programs should be tailored to the patient’s baseline motor status, stage of stroke recovery, and specific functional goals.

Future research should aim to establish standardized intervention protocols that reflect stroke phase and severity while also incorporating longer follow-up periods to assess the sustainability of treatment effects. It is also essential to evaluate broader outcomes such as quality of life and community reintegration, which reflect the real-world impact of RAGT beyond motor function. In addition, economic analyses are needed to assess cost-effectiveness and guide equitable resource allocation in clinical and policy contexts.

In conclusion, RAGT represents a promising and evidence-based rehabilitation strategy that not only enhances motor recovery but also promotes autonomy and patient-centered care. Its thoughtful integration into personalized rehabilitation pathways has the potential to address the growing needs of aging stroke populations and improve the overall quality and efficiency of neurorehabilitation services.

## Figures and Tables

**Table 1:** General characteristics of included studies.

Author (Year) [Ref No.]	Experimental Group						Control Group					Outcome Measures
	Participants (n) (Male/Female)	Type of Stroke	Age (Years) Mean (SD)	Time Since Onset (Days) Mean (SD) (Classification)	Robot Type	Intervention	Participants (n) (Male/Female)	Type of Stroke	Age (Years) Mean (SD)	Time Since Onset (Days) Mean (SD) (Classification)	Intervention	
Belas dos Santos et al. (2018) [24]	7 (5/2)	I: 2 H: 5	44.40 (12.70)	1752.00 (335.80) (chronic)	Lokomat 5.0 (Hocoma AG, Volketswil, Switzerland)	CT (60 min/day, 2 days/week, 20 weeks) + RAGT (60 min/day, 1 day/week, 20 weeks)	8 (6/2)	I: 2 H: 6	56.40 (11.80)	3832.50 (1971.00) (chronic)	CT (60 min/day, 2 days/week, 20 weeks) + CT (60 min/day, 1 day/week, 20 weeks)	BBS TUG FIM
Calabrò et al. (2018) [25]	20 (12/8)	NR	69.00 (4.00)	300.00 (90.00) (chronic)	Ekso (Ekso Bionics, Richmond, CA, USA)	CT (60 min/day, 5 days/week, 8 weeks) + RAGT (45 min/day, 5 days/week, 8 weeks)	20 (11/9)	NR	67.00 (6.00)	330.00 (90.00) (chronic)	CT (60 min/day, 5 days/week, 8 weeks) + CT (45 min/day, 5 days/week, 8 weeks)	10 MWT RMI TUG
Chang et al. (2012) [26]	20 (13/7)	I: 12 H: 8	55.50 (12.00)	16.10 (4.90) (subacute)	Lokomat (Hocoma AG, Volketswil, Switzerland)	CT (60 min/day, 5 days/week, 2 weeks) + RAGT (40 min/day 5 days/week, 2 weeks)	17 (10/7)	I: 11 H: 6	59.70 (12.10)	18.20 (5.00) (subacute)	CT (60 min/day, 5 days/week, 2 weeks) + CT (40 min/day, 5 days/week, 2 weeks)	FMA-LE FAC
Geroin et al. (2011) [27]	10 (6/4)	NR	63.30 (6.40)	801.00 (153.00) (chronic)	Gait Trainer (GT1 Reha-Stim, Berlin, Germany)	CT (30 min/day, 5 days/week, 2 weeks) + RAGT (20 min/day 5 days/week, 2 weeks)	10 (9/1)	NR	61.10 (6.30)	807.00 (174.00) (chronic)	CT (30 min/day, 5 days/week, 2 weeks) + CT (20 min/day, 5 days/week, 2 weeks)	6 MWT 10 MWT FAC RMI MI
Han et al. (2016) [28]	30 (17/13)	I: 17 H: 3	67.89 (14.96)	21.56 (7.98) (subacute)	Lokomat (Hocoma AG, Volketswil, Zürich, Switzerland)	CT (30 min/day, 5 days/week, 4 weeks) + RAGT (30 min/day, 5 days/week, 4 weeks)	26 (15/11)	I: 16 H: 10	63.20 (10.62)	18.10 (9.78) (subacute)	CT (30 min/day, 5 days/week, 4 weeks) + CT (30 min/day, 5 days/week, 4 weeks)	K-MBI BBS FAC FMA-L
Husemann et al. (2007) [29]	16 (11/5)	I: 12 H: 4	60.00 (13.00)	79.00 (56.00) (subacute)	Lokomat (Hocoma AG, Volketswil, Zürich, Switzerland)	CT (30 min/day, 5 days/week, 4 weeks) + RAGT (30 min/day, 5 days/week, 4 weeks)	14 (10/4)	I: 10 H: 4	57.00 (11.00)	89.00 (61.00) (subacute)	CT (30 min/day, 5 days/week, 4 weeks) + CT (30 min/day, 5 days/week, 4 weeks)	FAC BI
Kelley et al. (2013) [30]	11 (7/4)	NR	66.91 (8.50)	1354.15 (chronic)	Lokomat (Hocoma AG, Volketswil, Zürich, Switzerland)	CT (30 min/day, 5 days/week, 8 weeks) + RAGT (35–40 min/day, 5 days/week, 8 weeks)	9 (6/3)	NR	64.33 (10.91)	525.6 (chronic)	CT (30 min/day, 5 days/week, 8 weeks) + CT (35–40 min/day, 5 days/week, 8 weeks)	10 MWT 6 MWT FMA-LE BI
Kim et al. (2019) [12]	25 (20/5)	I: 14 H: 11	57.70 (12.90)	60.00 (72.00) (subacute)	Morning Walk (CUREXO, Seoul, Republic of Korea)	CT (60 min/day, 5 days/week, 3 weeks) + RAGT (30 min/day 5 days/week, 3 weeks)	23 (13/10)	I: 18 H: 5	60.40 (13.20)	78.00 (93.00) (subacute)	CT (60 min/day, 5 days/week, 3 weeks) + CT (30 min/day 5 days/week, 3 weeks)	FAC 10 MWT MBI RMI BBS
Lee et al. (2023) [1]	26 (15/11)	I: 20 H: 6	63.04 (15.69)	27.90 (20.70) (subacute)	Morning Walk (CUREXO, Seoul, Republic of Korea)	CT (90 min/day, 5 days/week, 4 weeks) + RAGT (30 min/day 5 days/week, 4 weeks)	23 (11/12)	I: 20 H: 3	64.78 (12.81)	27.30 (21.30) (subacute)	CT (90 min/day, 5 days/week, 4 weeks) + CT (30 min/day, 5 days/week, 4 weeks)	FAC RMI 10 MWT BBS MBI
Louie et al. (2021) [31]	19 (16/3)	I: 12 H: 7	59.60 (15.80)	36.70 (19.0) (subacute)	EksoGT (Ekso Bionics, Richmond, CA, USA)	CT (25%) (45–60 min/day, 1–2 days/week, 8 weeks +RAGT(75%) (60 min/day, 3 days/week, 8 weeks)	17 (10/7)	I: 13 H: 4	55.30 (10.60)	40.90 (19.80) (subacute)	CT (100%) (45–60 min/day, 4–5 days/week, 8 weeks)	FAC 6 MWT FMA-LE BBS
Morone et al. (2011) HM [32]	12 (8/4)	I: 9 H: 3	68.33 (9.11)	21.92 (10.72) (subacute)	Gait Trainer (GT Reha-Stim, Berlin, Germany)	CT (140 min/day, 5 days/week, 4 weeks) + RAGT (20 min/day, 5 days/week, 4 weeks)	12 (7/5)	I: 12 H: 0	62.92 (17.43)	20.00 (16.58) (subacute)	CT (140 min/day, 5 days/week, 4 weeks) + CT (20 min/day, 5 days/week, 4 weeks)	FAC MI BI 10 MWT
Morone et al. (2011) LM [32]	12 (7/5)	I: 9 H: 3	55.58 (13.35)	16.25 (11.33) (subacute)	Gait Trainer (GT Reha-Stim, Berlin, Germany)	CT (140 min/day, 5 days/week, 4 weeks) + RAGT (20 min/day 5 days/week, 4 weeks)	12 (6/6)	I: 11 H: 1	60.17 (9.59)	20.00 (12.76) (subacute)	CT (140 min/day, 5 days/week, 4 weeks) + CT (20 min/day, 5 days/week, 4 weeks)	FAC MI BI 10 MWT
Morone et al. (2018) [33]	50 (35/15)	I: 38 H: 12	61.90 (11.94)	19.30 (14.30) (subacute)	Gait Trainer (GT Reha-Stim, Berlin, Germany)	CT (180 min/day, 5 days/week, 2 weeks) + RAGT (40 min/day 5 days/week, 2 weeks)	50 (31/19)	I: 43 H: 7	63.48 (12.93)	16.50 (11.20) (subacute)	CT (180 min/day, 5 days/week, 2 weeks) + CT (40 min/day 5 days/week, 2 weeks)	FAC BI
Ng et al. (2007) [34]	17 (11/6)	I: 13 H: 4	66.60 (11.30)	18.90 (8.40) (subacute)	Gait Trainer (GT Reha-Stim, Berlin, Germany)	CT (100 min/day 5 days/week, 4 weeks) + RAGT (20 min/day 5 days/week, 4 weeks)	21 (13/8)	I: 18 H: 3	73.4 (11.50)	17.50 (8.40) (subacute)	CT (100 min/day 5 days/week, 4 weeks) + CT (20 min/day 5 days/week, 4 weeks)	MI BBS FAC BI FIM
Ochi et al. (2015) [35]	13 (11/2)	I: 5 H: 8	61.80 (7.50)	22.9 (7.40) (subacute)	Gait-Assistance Robot (GAR)	CT (60 min/day, 5 days/week, 4 weeks) + RAGT (20 min/day 5 days/week, 4 weeks)	13 (9/4)	I: 5 H: 8	65.50 (12.10)	26.10 (8.00) (subacute)	CT (60 min/day, 5 days/week, 4 weeks) + CT (20 min/day, 5 days/week, 4 weeks)	FMA FAC 10 MWT FIM
Park et al. (2018) [36]	12 (7/5)	I: 9 H: 3	55.58 (10.42)	219.90 (33.00) (chronic)	Lokomat Pro (Hocoma AG, Zurich, Switzerland)	CT (30 min/day, 5 days/week, 6 weeks) + RAGT (45 min/day, 3 days/week, 6 weeks)	16 (9/7)	I: 7 H: 9	57.50 (9.90)	232.50 (53.10) (chronic)	CT (30 min/day, 5 days/week, 6 weeks) + CT (45 min/day 3 days/week, 6 weeks)	BBS TUG 10 MWT FMA MBI
Park et al. (2020) [37]	7 (-/-)	I: 7 H: 2	76.29	<14.00 (subacute)	Walkbot locomotor training, (P&S Mechanics, Seoul, Republic of Korea)	CT (60 min/day, 7 days/week, 2 weeks) + RAGT (30 min/day, 7 days/week, 2 weeks)	7 (4/3)	I: 7 H: 0	69.86	<14.00 (subacute)	CT (60 min/day, 7 days/week, 2 weeks) + CT (30 min/day, 7 days/week, 2 weeks)	BBS FAC
Peurala et al. (2005) [38]	15 (13/2)	I: 7 H: 8	51.20 (7.90)	876.00 (949.00) (chronic)	Gait Trainer (GT1 Reha-Stim, Berlin, Germany)	CT (55 min/day, 5 days/week, 3 weeks) + RAGT (20 min/day 5 days/week, 3 weeks)	15 (11/4)	I: 8 H: 7	52.30 (6.80)	1460.00 (2117.00) (chronic)	CT (55 min/day, 5 days/week, 3 weeks) + CT (20 min/day 5 days/week, 3 weeks)	10 MWT 6 MWT FIM
Pohl et al. (2007) [16]	77 (50/27)	I: 61 H: 16	62.30 (12.00)	29.40 (12.60) (subacute)	Electromechanical Gait Trainer (Reha-Stim, Berlin, Germany)	CT (20 min/day 5 days/week, 4 weeks) + RAGT (25 min/day 5 days/week, 4 weeks)	78 (54/24)	I: 63 H: 15	64.00 (11.60)	31.50 (13.30) (subacute)	CT (20 min/day 5 days/week, 4 weeks) + CT (25 min/day 5 days/week, 4 weeks)	FAC BI RMI MI
Taveggia et al. (2016) [39]	13 (7/6)	NR	71.00 (5.00)	60.10 (49.50) (subacute)	Lokomat (Hocoma AG, Zurich, Switzerland)	CT (60 min/day 5 days/week, 5 weeks) + RAGT (30 min/day 5 days/week, 5 weeks)	15 (10/5)	NR	73.00 (7.00)	39.40 (31.70) (subacute)	CT (60 min/day 5 days/week, 5 weeks) + CT (30 min/day 5 days/week, 5 weeks)	6 MWT 10 MWT FIM
Tong et al. (2006) [40]	15 (9/6)	I: 11 H: 4	66.10 (9.90)	18.90 (9.10) (subacute)	Gait Trainer (GT II Reha-Stim, Berlin, Germany)	CT (40 min/day 5 days/week, 4 weeks) + RAGT (20 min/day 5 days/week, 4 weeks)	20 (12/8)	I: 17 H: 3	71.40 (14.00)	18.90 (8.40) (subacute)	CT (40 min/day 5 days/week, 4 weeks) + CT (20 min/day 5 days/week, 4 weeks)	MI BBS FAC BI FIM
Wu et al. (2012) [41]	24 (15/9)	I: 9 H: 15	50.00 (12.00)	27.00 (7.00) (subacute)	AVATAR-02 (Shanghai Zhanghua Technology Development Company, Shanghai, China)	CT (45 min/day 6 days/week, 8 weeks) + RAGT (10∼20 min/day 6 days/week, 8 weeks)	24 (16/8)	I: 11 H: 13	49.00 (10.00)	26.00 (8.00) (subacute)	CT (45 min/day 6 days/week, 8 weeks) + CT (10∼20 min/day 6 days/week, 8 weeks)	FMA FAC MBI
Yoo et al. (2023) [42]	9 (4/5)	I: 7 H: 2	61.00	19.00 (subacute)	ExoAtlet Medy (ExoAtlet Asia Co., Ltd., Seoul, Republic of Korea)	CT (90 min/day 5 days/week, 4 weeks) + RAGT (30 min/day 3 days/week, 4 weeks)	8 (5/3)	I: 5 H: 3	65.00	43.00 (subacute)	CT (90 min/day 5 days/week, 4 weeks) + CT (30 min/day 3 days/week, 4 weeks)	FAC FMA-LE BBS TUG 10 MWT K-MBI

I = Ischemic stroke; H = Hemorrhagic stroke; NR = Not reported; 10 MWT = 10-Meter Walk Test; 6 MWT = 6-Minute Walk Test; BBS = Berg Balance Scale; BI = Barthel Index; FAC = Functional Ambulation Category; CT = Conventional Therapy; FIM = Functional Independence Measure; FMA = Fugl–Meyer Assessment; FMA-LE = Fugl–Meyer Assessment of Lower Extremity; K-MBI = Korean version of the Modified Barthel Index; MI = Motricity Index; RAGT = Robot-Assisted Gait Training; RMI = Rivermead Mobility Index; TUG = Timed Up and Go.

**Table 2:** Summary of meta-Analytic effect sizes and subgroup analysis results. This table presents the pooled standardized mean differences (SMDs) or mean differences (MDs) with 95% confidence intervals for primary outcomes (gait function, gait speed, balance, and ADLs), along with subgroup analyses by robot type, stroke chronicity, and intervention dose.

Outcome	Overall Effect Size (95% CI)	Subgroup 1	Subgroup 2	I^2^ (%)
Gait Function	SMD = 0.51 (0.21–0.81)	End-effector: 0.53	Exoskeleton: 0.55	73
Gait Speed	SMD = 0.47 (0.12–0.83)	Subacute: 0.31	Chronic: 0.76	62
Balance	MD = 4.58 (3.03–6.13)	–	–	2
ADLs	SMD = 0.35 (0.14–0.56)	≤15 sessions: High	>15 sessions: Moderate	39

## Data Availability

No new data were created or analyzed in this study.

## Supplementary Materials

The following supporting information can be downloaded at: https://www.mdpi.com/article/10.3390/jcm14134809/s1, Table S1: Summary of Outcome Measures, Scoring Direction, and Clinical Interpretation Used in the Meta-Analysis.
