# Supplementary material for: Effectiveness of Robot-Assisted Gait Training in Stroke Rehabilitation: A Systematic Review and Meta-Analysis
Source: J Clin Med. 2025 Jul 7;14(13):4809. doi: 10.3390/jcm14134809 (PMC12250684; doi:10.3390/jcm14134809)
Supplement: Supplementary file 1 [file jcm-14-04809-s001.zip › jcm-3685521-supplementary.pdf]

Supplementary Table S1. Summary of Outcome Measures, Scoring Direction, and Clinical Interpretation Used in the Meta-Analysis

Outcome	Range	Direction	Clinical Interpretation
Berg Balance Scale (BBS)	0–56	Higher = better	Balance control
Timed Up & Go (TUG)	seconds	Lower = better	Mobility, fall risk
Functional Independence Measure (FIM)	18–126	Higher = better	ADL independence
10-Meter Walk Test (10MWT)	m/s	Higher = better	Gait speed
